# Epidemiological analysis of injury occurrence and current prevention strategies on international amateur football level during the UEFA Regions Cup 2019

**DOI:** 10.1007/s00402-021-03861-9

**Published:** 2021-03-19

**Authors:** Dominik Szymski, Volker Krutsch, Leonard Achenbach, Stephan Gerling, Christian Pfeifer, Volker Alt, Werner Krutsch, Oliver Loose

**Affiliations:** 1 Department of Trauma Surgery, University Medical Centre Regensburg, Regensburg, Germany; 2 Department of Otorhinolaryngology, Paracelsus Medical University Nuernberg, Nuernberg, Germany; 3 Department of Trauma Surgery, University Medical Centre Wuerzburg, Wuerzburg, Germany; 4 Clinic of Paediatrics, Clinic St. Hedwig, Regensburg, Germany; 5 SportDocsFranken, Nuremberg, Germany; 6 Department of Orthopedic Surgery, Olga Hospital, Klinikum Stuttgart, Stuttgart, Germany

**Keywords:** Amateur, Football, Soccer, Tournament, Epidemiology, Prevention, Risk factors

## Abstract

**Introduction:**

Football is the most popular sport worldwide and results in a high frequency of injuries. So far, mainly injuries in professional football have been investigated, and the literature lacks data regarding detailed injury epidemiology and current prevention data in amateur football tournaments.

**Materials and methods:**

A prospective cohort study investigated an international amateur football tournament, the UEFA Regions’ Cup, which took place in 2019 in Germany. Injury epidemiology, current prevention strategies of the teams and the implementation of the UEFA concussion protocol were investigated in detail by means of standardized injury definitions and data samples for football (Fuller et al., Scand J Med Sci Sports 16:83–92, https://doi.org/10.1111/j.1600-0838.2006.00528.x, 2006).

**Results:**

138 player of 8 teams participated in this study, while 39 players were excluded. Overall injury incidence was 12.5 per 1000 h total football exposure, 43.5 per 1000 h for match exposure. No injuries were registered during training. Injury prevalence was 14.1% per player and 1.1 injuries per match were registered. The lower extremity was predominantly affected by injuries (71.4%) and the majority of injuries (78.6%) were non-severe injury types like contusions (50%) and sprains (18.2%). Two head injuries, one contusion and one skin lesion, were handled by the guidelines of the UEFA concussion protocol. 44.4% of the players indicated at least one previous injury before tournament, 45.3% of them during the last two football seasons before start of the tournament. Injury prevention performance was included in all participating teams during the tournament by warm up or training strategies (100%). During the warm-up program just 5 exercises of the FIFA 11 + program was detected by this investigation in participating teams to be done by more half of the teams. Running exercises were the most frequently performed exercises, while trunk muscle exercises were less represented (14.3%).

**Conclusion:**

This study presents for the first time epidemiological injury and prevention data of the UEFA Regions Cup. Injury incidence was higher compared to injury reports of regular seasons, but lower compared to other amateur football tournaments. Currently used prevention programs revealed trunk muscle exercises as often neglected.

## Introduction

Professional football (soccer) is one of the most popular types of sports worldwide [21], but the number of amateur players is even higher and includes millions of recreational players, participation is growing annually [10]. With this huge number of players, the number of injuries during football is steadily increasing. Yearly in Germany 2 million sport injuries occur and most of them are in relation to football, especially in amateur football [19, 29]. Although most of the players participate at a recreational level, existing epidemiological injury surveys mainly analyse professional football. Among these players, investigations for all types of competitions, such as seasonal injury studies or tournament research projects of international events, are available. In particular, the Olympic football event, UEFA Champions league, FIFA World Cup and European Championship are of particular special focus. Studies suggest that high physical and mental load during a short time burdens the athlete and is responsible for an increased rate of injuries [14, 18, 23–26, 44].

Though most of football players play at an amateur level, existing statistics are rare and mostly observing a whole season [6, 21]. A rarely investigated but common type of competition among this level of performance are tournaments [27, 28]. In epidemiological investigations a lower incidence of injuries among amateur players in comparison to professional athletes, especially in competitions, was verified [6, 21]. This is attributed to potential higher muscle strength and flexibility in professionals, as well as poor preparation in amateurs [27, 28, 31].

The UEFA Regions’ Cup is an international football tournament for amateurs that takes place biennially. It was introduced in 1999 and is the successor of the UEFA amateur cup, which was played between 1966 and 1978. All 55 UEFA member associations are invited to register one regional representative amateur team to the preliminary and intermediate rounds in order to qualify for the final tournament. The qualifying matches are held in two rounds and the teams contest for eight starting positions in the final event (https://www.uefa.com/regionscup/).

This unique championship of amateur level football at international level gives the opportunity to investigate the incidence of injuries. Aim of this study was to identify the incidence of injury at the final tournament, the prevalence of previous injuries of participating players with focus on localization and type as well as the examination and evaluation of warm-up program implementations. Furthermore, the established UEFA guidelines for players, doctors and referees concerning the management of concussion were investigated for secondary injury prevention.

## Methods

### Study population

**Fig. 1:**
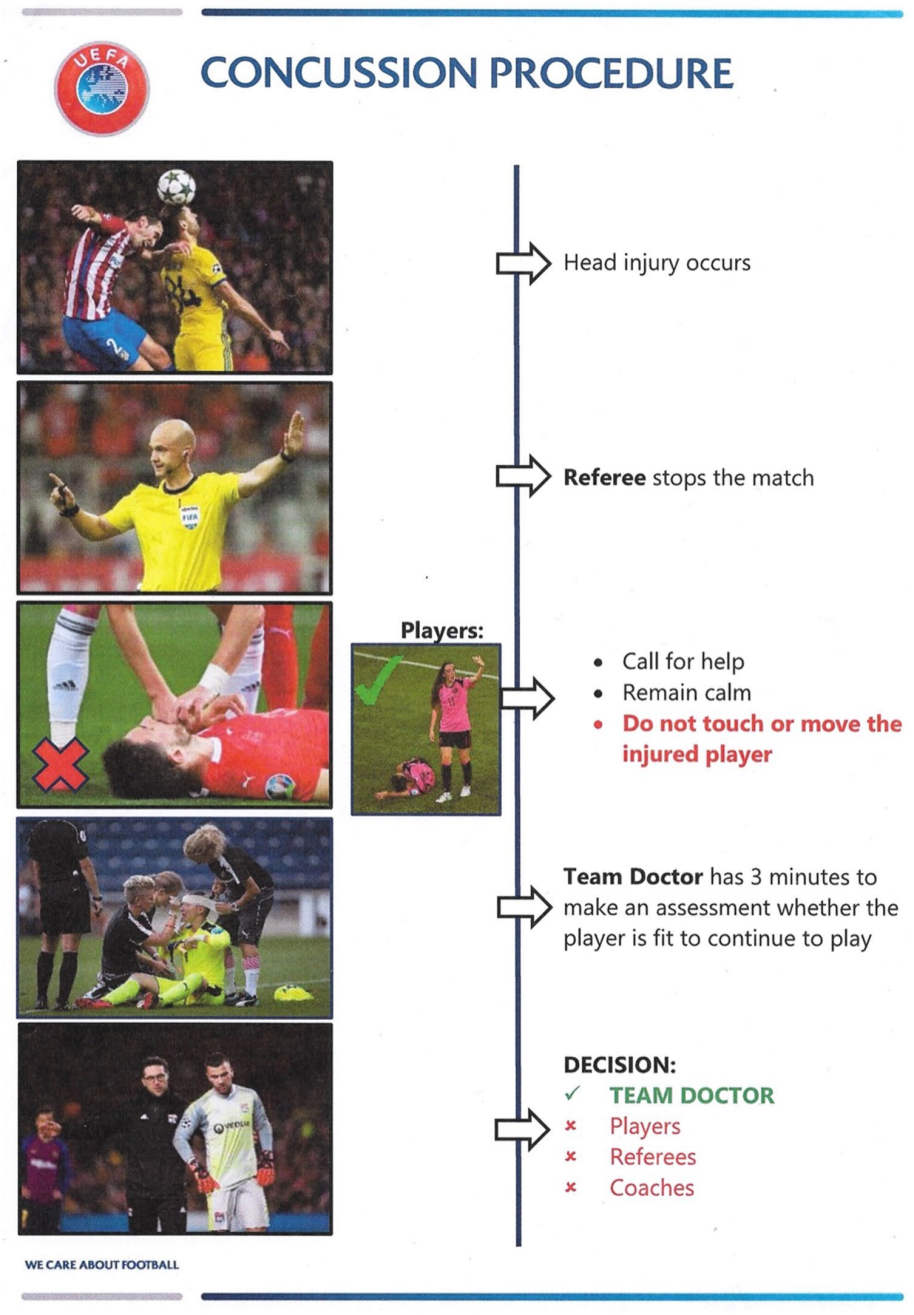
UEFA concussion management on field

This prospective cohort study analysed the final round of the UEFA Regions’ Cup, an international tournament for amateur football players. Regional representative teams from 39 UEFA member associations participated in the qualifying competition from which eight teams qualified for the final tournament, which was executed with two groups of, respectively, 4 teams. In the final match the winning teams of both groups compete against each other. The event took place from 18.06 to 26.06.2019 in Bavaria, where every team had to play at least three matches. During the tournament, data were collected pertaining to injuries during matches and previous injuries. We also investigated the warm-up program utilized by teams before official matches and compared it to the FIFA 11 + injury prevention program (https://www.fifamedicalnetwork.com). Each team medical supervisor was informed about this survey, including the aims as well as the purpose of the research, at the technical pretournament meeting. Players were briefed by the research team and a written consent was obtained from each participant. To address specifically the secondary prevention of head injuries, all participating amateur football teams were instructed for the guidelines of the UEFA protocol for concussion management. All team doctors were educated previous to the tournament in a specific meeting. Additionally, the hosting country provided bulletin in form a poster (Fig. 1), where the concussion guidelines were summarized. Each match of the tournament had a medical officer, who assessed both the warm-up program before the match and also the correct management of head injuries.

All players who participated in the final round of the UEFA Regions’ Cup 2019 were included into the study population. Missing consent or a missing injury report were exclusion criteria. The study was approved by the Ethics Committee of the University of Regensburg (ID: 11-167-1-101).

### Tournament rules

The UEFA Regions’ Cup lasts for 9 days with at least three matches per team in the group phase and one additional final match for the first-ranked teams of each group. According to the regulations of the European Football Association (UEFA) only amateur players, who have never competed on professional level were allowed to participate (https://www.uefa.com/regionscup/). The tournament and the matches were played according to the IFAB Laws of the Game (https://www.theifab.com). Between the first and the second group match one free day was scheduled, and between the second and third match two days of rest.

### Data collection

Data collection, inspired by the Consensus statement of Fuller et al. [15], consisted of a standardized demographic baseline questionnaire, including information about age, height, weight, experience in football as general and in international football. Additionally, data about player position, preferred kicking leg and medical history were recorded. Past injury information was categorized by year, type, side and treatment, and each injury classified as severe or not severe (Table 1) [15]. Furthermore, investigations of the warm-up program and correlation with exercises from the FIFA 11 + prevention program were performed.

**Table 1:** Injury classification of severity level adopted by Krutsch et al. [29]

Severe injury	Non-severe injury
Fracture	Skin lesion
Rupture	Sprain
Dislocation	Strain
Concussion	Contusion

The design and methods of the research are inspired by the UEFA guidelines for epidemiological studies [17]. Match and training exposure of all official events was registered and used in the calculation of incidence [15].

### Statistical analysis

Continuous data are expressed as mean ± standard deviation (SD) and categorical data as frequency counts (percentages). Incidence rates of overall injury were calculated by dividing the number of events by the total match exposure time as well as the training exposure in 1000 player hours relating to Fuller et al. [14]. Odds ratios and rate ratios accompanied by the corresponding 95% confidence interval (CI) are reported as effect estimates. Significance between previous and new injuries is calculated using the Chi test. The significance level was set to *p* < 0.05. All analyses were performed with IBM SPSS Statistics, version 26.0.

## Results

**Fig. 2:**
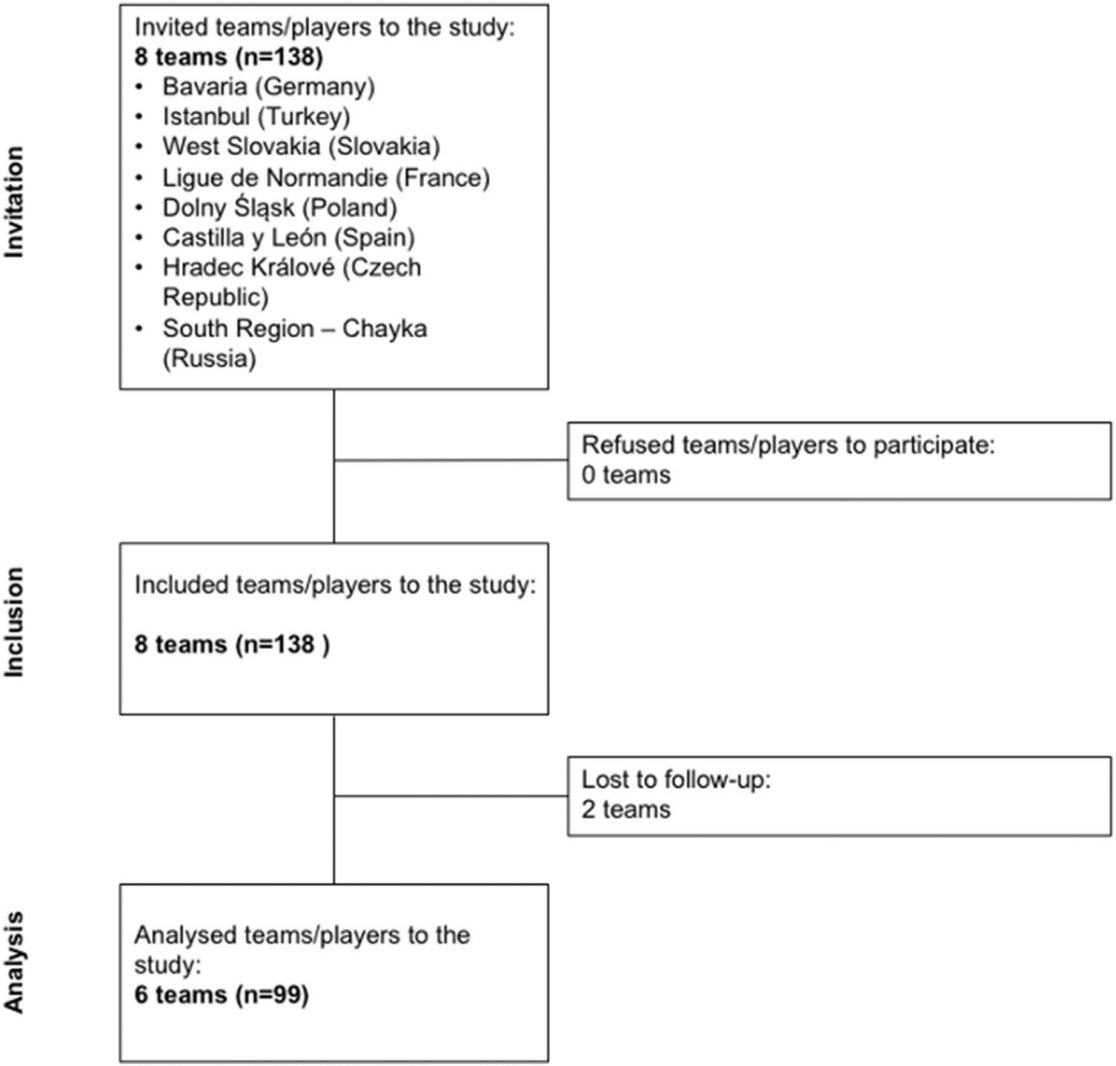
Flowchart of the study population

138 players from eight different teams were included into this study. Data of 99 players, from 6 teams, constituted the final group for analysis statistics; 39 players (28.3%) were excluded from this research due to missing or incomplete injury protocol (Fig. 2).

### Anthropometric and football-specific data

Study participants demonstrated an average age of 25.4 years with a height of 180.9 cm and a weight of 76.8 kg. Athletes had an average experience in football of 17.2 years. Mainly the players acted in the midfield (35.4%) or defence (32.3%). 84.5% of athletes were right leg dominant. Observing the exposure of the players in competition and exercise, we determined 4.88 h of match and 8.28 h during training per player (Table 2).

**Table 2:** Anthropometric and football-specific data of the study population

	Mean ± SD
Age (years)	25.4 ± 4.55
Height (cm)	180.9 ± 5.04
Weight (kg)	76.8 ± 6.94
BMI (kg/m^2^)	23.4 ± 1.54
Experience in football (years)	17.2 ± 5.14
Experience in international football (years)	2.3 ± 2.66

Position	*n* (%)
Goalkeeper	11 (11.1)
Defence	32 (32.3)
Midfield	35 (35.4)
Striker	21 (21.2)

Dominant leg	*n* (%)
Right leg	82 (84.5)
Left leg	13 (13.4)
Both legs	2 (2.1)

Exposure (in h)	Total (per player)
Match exposure	39 (4.875)
Training exposure	66.25 (8.28)

### Medical history

All included players (*n* = 99) combined had a total of 58 previous football-related injuries. More than half of the players (*n* = 55, 55.6%) declared no previous injuries in their football career. 35 athletes indicated one injury (35.4%) and 9 two injuries (9.1%), no player stated more than two injuries. 24 of the injuries (45.3%) occurred in the current and the season before (Table 3).

**Table 3:** Medical history of participating players

Number of previous Injuries	*n* (player in %)
0	55 (55.6)
1	35 (35.3)
2	9 (9.1)
58 injuries (total)	99 players

Year of previous Injury	*n* (injuries in %)
Current season	13 (33.3)
1 season before	11 (28.2)
2 seasons before	3 (7.7)
3 seasons before	3 (7.7)
4 seasons before	4 (10.3)
More than 4 seasons before	5 (12.8)
Surgery required	**4 (7.5)**

Main localization of all previous injuries was in the lower extremity (*n* = 22) followed by the upper extremity (*n* = 9). Fractures (*n* = 14; 26.4%), sprains (*n* = 10; 18.9%) and ruptures (*n* = 8; 15.1%) were the most common types of injuries. Classification of the lesions into severity resulted in 28 no severe (48.3%), 16 severe injuries (27.6%) and 14 injuries (24.1%) with insufficient data for declaration of severity. Therefore four lesions (7.5%) had to be treated surgically.

### Warm-up program

**Fig. 3:**
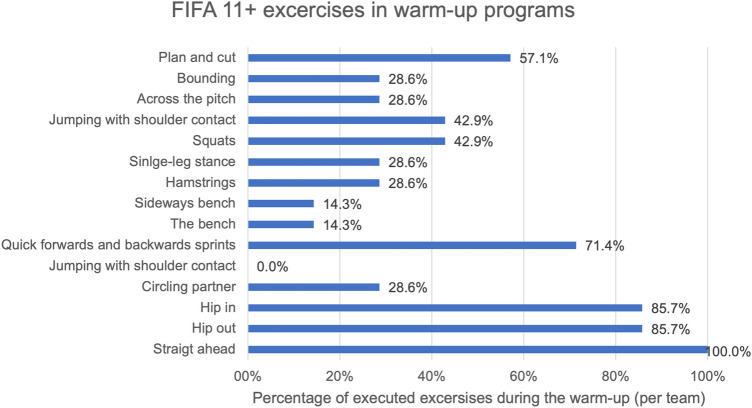
Percentage of exercises in the warm-up program during the UEFA Regions' Cup

Warm-up data of one team were missing. Out of 15 exercises of the FIFA 11 + prevention program, only five were performed by more than half of the teams. These were “straight ahead” (100%), “hip in” (85.7%), “hip out” (85.7%), “quick forwards and backwards sprints” (71.4%) and “plan and cut” (57.1%) (Fig. 3). There was no significant correlation between injury incidence in the tournament and the number of elements of the FIFA 11 + program performed for warm-up (*p* > 0.05).

### Injury profile during the tournament

**Fig. 4:**
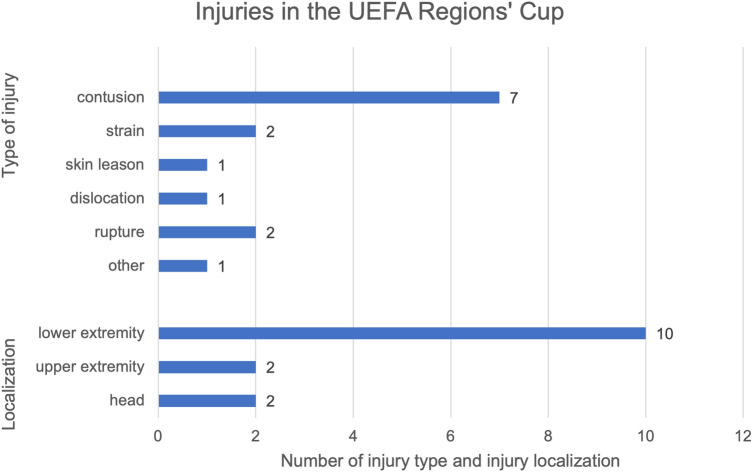
Number of injuries during the tournament with regard to localization and type

In total 14 injuries appeared during the UEFA Regions’ Cup, which results in an incidence of 43.5 injuries per 1000 h of match exposure and a prevalence of 14.1% per player during the tournament. We determined an incidence of 12.5 injuries in 1000 h total football experience (match and training). This results in 1.1 injuries per match during this event. No training injuries were registered during the tournament. The recorded injuries mainly occurred in the second match (50%) and 7 affected the left side (50%). The predominant site of injury was the lower extremity (*n* = 10), with a share of 71.4%. Main pathologies were the occurrence of contusions (*n* = 7), followed by strains (*n* = 2) and ruptures (*n* = 2) (Fig. 4). Half of the injuries were classified as time loss injury with average 11.57 days off from active training, whereby 21.4% were ranked as severe (Table 4). There was no significant correlation between previous injuries and injuries during the tournament (*p* = 0.897).

**Table 4:** Injuries in UEFA regions' cup tournament

Time of injury (match number)	*n* (%)
1	5 (35.7)
2	7 (50.0)
3	2 (14.3)
4 (final)	0 (0.0)

Side	*n* (%)
Right	4 (30.8)
Left	7 (53.8)
No definition possible (head injury)	2 (15.4)
Time loss injury	7 (50.0)

Days out of training	*n* (%)
0	6 (46.1)
1–3	4 (30.8)
4–7	1 (7.7)
8–28	1 (7.7)
> 28	1 (7.7)

The management of two head injuries (one contusion, one skin lesion), where medical treatment on field was necessary, was performed completely accurately corresponding to the recommended concussion protocol.

## Discussion

The main finding of the study was a high incidence of injuries during this international amateur football tournament, which represents the first scientific report of this kind of amateur football event. With regard to injury prevention strategies, the participating teams showed major deficits with regard to known injury prevention warm-up exercises, whereas the UEFA concussion protocol was always adhered to.

Tournament injury reports showed in previously published research a higher injury incidence than seasonal reports, which was until now only published for professional football [7, 14, 23–26, 36]. The injury frequency in this football tournament with 43.5/1000 h is four times higher compared to a seasonal injury report in male elite football with an incidence of 9.7/1000 h [35]. Greater physical and especially mental load on the athletes within football events, as well as cumulative number of matches, which themselves are already correlated with a higher risk of injury, are the postulated causative factors [2, 29, 32]. Herewith the injury incidence in this amateur football event is lower than the overall incidence in the first and second professional league in Germany which was last given as 50.2/1000 h football. [6, 12, 21, 36, 38].

Amateur football seasons showed generally lower injury incidences. In Spanish amateur football during one season 1.15 injuries per 1000 h match exposure were registered, whilst Peterson et al. found an incidence of 2.1 injuries in 1000 h of football among amateurs [21, 40]. Other investigations of amateur football in tournaments showed an even higher incidence, which was calculated between 469 and 832 injuries in 1000 h of match exposure for overuse and traumatic injuries [27, 28]. Thus, these amateur tournaments represented recreational student events and not high-level organized football like in the UEFA Regions Cup. The teams and players in the UEFA Regions’ cup represented an international community of amateur teams, playing on a high level below the professional football stage.

The lower extremity was reported as most affected injury in this tournament, which aligns with previous scientific literature [3, 11, 19, 21, 27, 29]. The injury survey recorded previous injuries in the participating players of the tournament. Previous injuries have been demonstrated to be the most important risk factor for further injuries [13, 37, 45]. Our study showed a previous injury in 33.3% of players without correlation to injuries that occurred at the tournament. This rate of previous injuries shows that injuries are a continuous problem in amateur football, while this prevalence of 33.3% of injured players is significantly lower than normal injury prevalence over one complete season, which is up to 80% [32]. Most important consequence to prevent re-injuries is to respect healing time and avoid too early return to play after injuries. Compared to the previous literature, the UEFA Regions Cup shows a clear trend to non-severe injuries. 36.4% of previous injuries before the tournament were severe injury types in the participating players, and during the tournament only 21.4%. Compared to current literature, this study reveals a similar percentage of non-severe injury types like sprains, strains or contusions [11, 21, 27, 40].

To prevent injuries in football, sport-specific and multimodal concepts are necessary to include all actors in the prevention strategies [30, 43]. Two of the main actors in injury prevention, players and coaches, have a high interest in this subject and believe that injuries in football are a severe problem. Regular physiotherapy and screening examinations are considered to be important, whereas warm-up prevention programs like FIFA 11 + are insufficient known by coaches and little by amateur players [34]. A sufficient preparation by warm-up or training exercises is essential [8, 42]. Otherwise, deficits in preparation and injury prevention strategies are well-described as important fundament for higher injury incidence [8, 20, 27]. Implementation of a structured injury prevention program has been sufficiently investigated and result in significant reduction of injuries up to 50% of all injuries in a running season [5, 8, 33, 39, 41]. Especially FIFA 11 + provides a high number of valid publications with high level of evidence for preventive effects in case of frequent usage. Participating amateur teams of the UEFA Regions Cup showed in their performed warm-up programs during training and match exposure during the tournament only few elements of established programs like FIFA 11 + . Just 5 of 15 preventive exercises of the FIFA 11 + program were performed by at least half of the participating teams (4 of 7 teams). While running exercises were as frequent as performed by the UEFA Regions Cup teams like in FIFA 11 + , other essential preventive exercises were almost lacking. This agrees with the findings that football-specific injury prevention exercises are known in warm-up programs, but the transfer to the daily routine is lacking [34]. One of these missing exercises are trunk muscle exercises to improve trunk muscle stability, which is well-described with effect on injury prevention in previous literature [5, 22]. This lack of strength and flexibility in the trunk capacities of amateur football players was already reported in previous literature [31]. In order to improve injury prevention in all amateur football levels, sufficient warm-up and training programs with specific focus on neuromuscular exercises on trunk and leg axis are an essential part for training and match preparation and the improvement of the implementation of these exercises is an important point for sustainability in injury prevention [1, 4, 5, 20, 32]. Therefore, amateur football tournaments on international level, e.g., the UEFA Regions Cup, represent a perfect platform to spread knowledge about efficacy of warm-up programs for participating teams.

As part of the secondary prevention strategies, the UEFA provides specific guidelines and medical concepts for the teams. Especially for the management of concussion the UEFA installed a bulletin (Fig. 1) in each locker room of the playing teams, which summarized the principles of the recommended concussion management. In this study population there were two slight head injuries, where the team doctors performed exactly the educated guidelines. Head injuries are generally often trivialized and therefore they are an important part of prevention strategies in football associations [29, 36]. The education of strategies of secondary prevention in head injuries is essential and should be an integral part of future practical routine in amateur football, also over beyond UEFA or FIFA tournaments because of its proven evidence [9, 16].

Limitations of this study were the small number of participants, which can be explained by the small tournament size of 8 teams. In addition, 2 of the 8 teams were excluded due to missing data, which was atypical drop-out rate for this playing level [27, 28, 32]. The prospective design, standardized methods and data collection the unique investigation of injury data and analysis of prevention deficits are strengths of this study.

## Conclusion

This study presents for the first time epidemiological injury and prevention data of the UEFA Regions Cup. Injury incidence was higher compared to injury reports of regular seasons, but lower compared to other amateur football tournaments. Currently used prevention programs revealed trunk muscle exercises as often neglected.
